# Looking behind the Solubilization Curtain: Microdialysis Provides Near-Real-Time Noncolloidal Drug Concentrations during In Vitro Lipolysis

**DOI:** 10.1021/acs.molpharmaceut.5c00640

**Published:** 2025-10-03

**Authors:** Mikkel Højmark Tønning, Annette Bauer-Brandl, Martin Brandl, Felix Paulus, Ann-Christin Jacobsen

**Affiliations:** † Department of Physics, Chemistry & Pharmacy, 6174 University of Southern Denmark, 5230 Odense, Denmark; ‡ Department of Pharmaceutics and Biopharmaceutics, 9179 Kiel University, 24118 Kiel, Germany

**Keywords:** Indomethacin, medium-chain lipids, LBF type IIIB, SEDDS, digestion, biopharmaceutics

## Abstract

Lipid-based formulations can enhance the oral absorption of poorly water-soluble drugs. Their performance is typically evaluated by in vitro lipolysis. For this, samples are usually prepared by centrifugation, and formulation performance is evaluated based on the concentration in the aqueous phase. However, several studies have questioned the predictiveness of the in vitro lipolysis method. A reason for the in vitro-in vivo mismatch may be that centrifugation cannot separate truly dissolved drug molecules from molecules associated with colloidal assemblies such as mixed micelles. The present study tested microdialysis as an alternative sampling technique for in vitro lipolysis by which truly dissolved drug molecules (i.e., the free fraction) can be separated from colloid-associated drug molecules. Thereby, a better mechanistic understanding of the formulation performance will possibly be achieved. Indomethacin and a medium-chain type IIIB lipid-based formulation were used as model drug and model formulation, respectively. Microdialysis sampling was found compatible with lipolysis medium with bile salts, phospholipids, and pancreatic enzymes. In a proof-of-concept study, microdialysis provided near-real-time concentrations of free indomethacin during the in vitro lipolysis process and revealed supersaturation of indomethacin. However, indomethacin supersaturation was also observed under nonlipolytic conditions. Based on microdialysis data, digestion would not influence the formulation performance. In contrast, conventional samples showed that lipolysis significantly decreased the solubilization capacity of the formulation. Other examples have been described in the literature where oral absorption from lipid-based formulations had been independent of digestion even though data from in vitro lipolysis with conventional sampling indicated the opposite. Overall, microdialysis is a promising and complementary sampling technique for the evaluation of lipid-based formulations by in vitro lipolysis.

## Graphical abstract

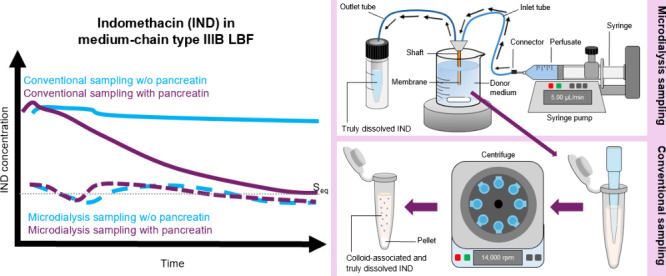

## 1 Introduction

Due to the increase of poorly water-soluble drugs during the past two decades,^1^ candidate-enabling oral formulations have come into focus in oral drug delivery research. One example are lipid-based formulations (LBFs), which are a promising choice for “grease-ball”-like molecules of intermediate to high lipophilicity (logP 4 to 5).^2^ LBFs contain oils, surfactants, and cosolvents and are classified according to the Lipid Formulation Classification System (LCFS) according to the fraction of each excipient class and the hydrophilicity of the surfactant(s). The LCFS classification spans from pure oil-based systems (type I), over type II LBFs that also contain a lipophilic surfactant, over type IIIA LBF with a hydrophilic surfactant, over type IIIB LBFs with a decreasing oil content, to type IV LBFs that are only composed of cosolvent(s) and surfactant(s).^3,4^

Upon oral administration, the majority of LBFs are subject to lipolysis (digestion) in the presence of gastric and pancreatic lipase, producing free fatty acids that form mixed micelles and other colloidal species with bile components, which keep poorly water-soluble drugs in solution.^5^ This digestion process can be crucial for enhancing the bioavailability of poorly water-soluble drugs using LBFs, especially for the most lipophilic type I and II formulations,^6,7^ whereas this was shown to be less important for the more hydrophilic LBF types.^8^

In vitro lipolysis, a modified dissolution method where the digestion process is mimicked by the addition of lipolytic enzymes, has become a common tool for the in vitro biopharmaceutical characterization and performance ranking of LBFs.^9^ During the experiment, the digestion of the formulation and the drug distribution between different phases is monitored. Here, digestion is monitored by titration of released free fatty acids with sodium hydroxide, and the drug distribution between different phases is monitored after separation commonly via centrifugation, e.g., benchtop centrifugation or ultracentrifugation, followed by quantification of the drug in the obtained phases.^10^

A typical in vitro lipolysis sample contains after ultracentrifugation up to 4 layers/phases: A pellet phase, an aqueous or micellar phase, an interphase, and an oil layer.^10,11^ When comparing benchtop and ultracentrifugation, the interphase was only present in samples that were separated via ultracentrifugation.^10^ Furthermore, it is more difficult to isolate the oil phase in samples separated via benchtop centrifugation as compared to ultracentrifugation.^10^ Of these up to 4 layers/phases, the drug concentration in the aqueous or micellar phase would commonly be used to evaluate the performance of the LBF. Recently, frequent in vitro in vivo mismatches have been observed when conducting in vitro lipolysis questioning the usefulness of the method.^9,12^

Several alterations have been tested to improve the predictability of the in vitro lipolysis model. The most frequently used protocol takes only intestinal digestion into account. However, gastric digestion contributes to around 20% of overall lipolysis.^13^ Therefore, one possible refinement of the in vitro lipolysis method is including a gastric digestion step at gastric pH and with gastric lipase as source of enzyme.^14^ Another approach is combining in vitro lipolysis with permeation.^15^ An in vitro lipolysis-permeation setup consists of a donor compartment containing the LBF in a lipolytic environment and an acceptor compartment, which are separated by a permeation barrier. There are various set-ups described in literature using different devices (e.g., small ones such as 96-well plates enabling high throughput^16^ or bigger ones such as the enabling absorption device^17^ ), and using different permeation barriers (e.g., tissue-based ones^18,19^ , cell-based ones,^17^ biomimetic ones^15,20^ or dialysis membrane ones^19^). In all these combined approaches, primarily the permeated drug concentration (i.e., in the acceptor) would be used for evaluating the performance of the LBF. To some extent, samples can also be taken the conventional way (i.e., from the donor) to yield additional information on formulation performance. However, it needs to be considered that sampling always disturbs the system in some way. Combined in vitro lipolysis-permeation seems, indeed, to give a better alignment with in vivo results.^17,21,22^

One hypothesis explaining why combined in vitro lipolysis-permeation yields a better in vitro-in vivo correlation is that primarily the fraction of drug that is truly dissolved (i.e., single drug molecules surrounded by a hydration shell, molecular dimers or multimers up to very small nanoparticles^23^ ) can (passively) permeate biomimetic or biological barriers, and enter the bloodstream in vivo.^24^ The drug concentration of the aqueous phase obtained through conventional lipolysis sample preparation comprises at least two drug fractions, a fraction of truly dissolved drug, and a fraction of drug solubilized within micelles or other colloidal assemblies small enough to resist being spun down during centrifugation. In contrast, the cumulative permeated amount of drug obtained from combined in vitro lipolysis-permeation experiments, although reflecting the fraction of permeable drug, does not allow back-calculation of the drug that is molecularly dissolved during lipolysis because there is an unknown delay between release and permeation. Thus, the permeated drug concentrations do not provide time-resolved information about concentrations of the molecularly dissolved drug in the donor. Therefore, combined in vitro lipolysis-permeation can be regarded as a “black-box system”.

Time-resolved concentrations of molecularly dissolved drug during in vitro lipolysis are crucial information to understand the LBF performance mechanistically. Until now, only ultracentrifugation could possibly yield this information. However, ultracentrifugation is rarely used in dissolution experiments, since it is a relatively challenging and time-consuming technique, and the instruments come at a high cost.

Recently, microdialysis sampling, which historically has been used for sampling in tissue, e.g., brain,^25^ was introduced as a method to separate truly dissolved drug molecules from colloid-associated drug molecules during dissolution of enabling drug formulations.^26–28^ The heart of a microdialysis setup are the microdialysis probes, which are needle-shaped devices with a probe tip that is semipermeable. For microdialysis sampling during, e.g., dissolution testing, those probes are inserted into the dissolution medium with the analyte of interest. Perfusion medium is then continuously pumped through the microdialysis probes via a syringe pump with suitable tubing. During perfusion, only the truly dissolved fraction of the analyte can pass the microdialysis membrane and enter the dialysate, whereas larger assemblies stay behind in the dissolution vessel. It needs to be mentioned that microdialysis is a nonequilibrium method and hence only can yield absolute values of the truly dissolved drug fraction after careful calibration, i.e., determination of a correction factor, which is also called recovery. Provided that the microdialysis probes have been calibrated carefully and are compatible with the medium, the free drug concentration can be followed almost in real time by microdialysis sampling.

In the present proof-of-concept study, we have investigated whether microdialysis sampling can be used to determine the truly dissolved drug concentrations occurring during in vitro lipolysis, exemplified on a medium-chain type IIIB LBF with the poorly water-soluble BCS class II drug indomethacin.^29^ Overall, this is an important first step to evaluate if microdialysis sampling may be able to improve the predictability of the in vitro lipolysis method for the performance evaluation of LBFs. At the same time, time-resolved and absolute concentrations of truly dissolved drug molecules during in vitro lipolysis, possibly obtained through microdialysis sampling, may improve the mechanistic understanding of LBF performance.

## 2 Materials and Methods

### 2.1 Chemicals

The model drug used in this study, indomethacin (IND), was purchased from Sigma-Aldrich Denmark ApS (Bro̷ndby, Denmark). From this supplier were also trifluoroacetic acid, CaCl_2_·2H_2_O, NaH_2_PO_4_·2H_2_O, tris-maleate, pancreatin from porcine pancreas (8 × USP specifications), polysorbate 80 (PS80), Cremophor EL (PEG-35 castor oil), and Carbitol (diethylene glycol monoethyl ether). NaCl and methanol (HPLC-grade) were from VWR International A/S (So̷borg, Denmark). NaOH pellets were from Merck A/S (Hellereup, Denmark). Capmul MCM CP EP/NF (a mixture of monoglycerides of caprylic and capric acid) was kindly donated by ABITEC corporation (Columbus, USA). Labrafac lipophile WL 1349 (a mixture of medium-chain triglycerides of caprylic and capric acid) was kindly donated by Gattefossé (Saint-Priest, France). FaSSIF/FeSSIF/FaSSGF powder comprising lecithin and sodium taurocholate was purchased from biorelevant.com (London, UK). All buffers were prepared using highly purified water from a Milli-Q reference A+ water purification system (Merck KGaA, Darmstadt, Germany) and salts of analytical grade.

### 2.2 Media Preparation

For in vitro lipolysis, a strong tris-maleate buffer was prepared according to Hedge et al.^30^ The buffer, which will be referred to as lipolysis buffer, consisted of 200 mM tris-maleate, 150 mM NaCl, and 1.4 mM CaCl_2_·2H_2_O. The buffer pH was adjusted to 6.5 with diluted NaOH. Based on the lipolysis buffer, lipolysis medium was prepared by adding 2.24 g/L FaSSIF/FeSSIF/FaSSGF powder to lipolysis buffer yielding a final concentration of 3 and 0.75 mM for sodium taurocholate and lecithin, respectively. Lipolysis medium was allowed to stand at room temperature for 2 h prior to use and was used the same day. Additionally, two alternative simulated intestinal media with sodium taurocholate and lecithin were prepared. These media contained either 1.5 mM sodium taurocholate and 0.375 mM lecithin or 15 mM sodium taurocholate and 3.75 mM lecithin, which corresponds to 0.5- and 5-times the sodium taurocholate and lecithin concentrations of lipolysis medium.

### 2.3 Preparation of Medium-Chain Type IIIB LBF Loaded with Indomethacin

A medium-chain type IIIB LBF loaded with indomethacin was prepared as described by Alskär et al.^29^ The composition is given in Table 1. Briefly, the liquid excipients (Cremophor EL, Carbitol, and Labrafac lipophile WL 1349) were preheated to 37 °C and weighed into vials. Capmul MCM CP EP/NF was melted at 60 °C before it was weighed into the vials. Subsequently, 42 mg indomethacin were added to the vials, the LBF was vortexed, and then incubated at 37 °C in a shaking water bath overnight. The drug loading corresponded to 80% of indomethacin’s solubility in the LBF according to Alskär et al.^29^

**Table 1:** Composition of the Medium-Chain Type IIIB Lipid-Based Formulation^a^

Excipient	Excipient type	Amount used [mg]	% (w/w)
PEG-35 castor oil (Cremophor EL)	Surfactant	312.50	50.0
Diethylene glycol monoethyl ether (Carbitol)	Cosolvent	156.25	25.0
Monoglycerides of caprylic and capric acid (Capmul MCM CP EP/NF)	Medium-chain monoglyceride	78.125	12.5
Triglycerides of caprylic and capric acid (Labrafac lipophile WL 1349)	Medium-chain triglyceride	78.125	12.5

a Indomethacin loading was 63 mg/g.

### 2.4 Quantification of Indomethacin by HPLC-UV

Indomethacin was quantified by HPLC-UV. The quantitative method has been described before.^23^ Briefly, the system was a Waters Alliance 2695D HPLC with a Kinetex XB-C18 column (4.6 × 150 mm, 3.5 μm), which was kept at 40 °C during analysis. Indomethacin was detected with a single-wavelength UV-detector at 256 nm. The mobile phase was 75:25 methanol:water with 0.1% (v/v) trifluoroacetic acid (all of HPLC-grade) and the flow rate was 1.0 mL/min. For analysis, 20 μL sample were injected into the system. The quantification method was linear in the ranges of 0.011–11 and 0.326–1060 μg/mL with limits of detection and limits of quantification being 0.003 and 0.009 μg/mL for the former concentration range and 0.44 and 1.33 μg/mL for the latter concentration range.

### 2.5 Determination of Equilibrium Solubility

Indomethacin’s solubility in lipolysis medium was determined in a shake-flask solubility study as previously described.^23^ Briefly, excess amount of indomethacin was added to 3 mL of lipolysis medium and incubated at 37 °C in a shaking water bath (*n* = 3). Solubility was determined after 24, 48, and 72 h of incubation. For this, samples were centrifuged at 14,000 rpm (24,104 × g) for 15 min at 37 °C using a Centrifuge 5804 R with a F-45-30-11 rotor (Eppendorf AG, Hamburg, Germany). The supernatant was filtered through a 20 nm Anotop syringe filter (Whatman, Maidstone, UK). After the first three droplets were discarded, 50 μL of the filtrate was diluted with 950 μL of HPLC mobile phase, and finally, indomethacin was quantified by HPLC-UV as described above.

### 2.6 Testing Microdialysis Sampling in Simulated Intestinal Conditions

For testing of microdialysis sampling of indomethacin in simulated gastrointestinal conditions, concentric CMA/20 microdialysis probes with polyethersulfone dialysis membrane tips of 30 mm effective length and 100 kDa molecular-weight cutoff (MWCO) pore size (CMA Microdialysis AB, Sweden) were used. The microdialysis probes were connected to 2.5 mL glass syringes, which were placed in a CMA/4004 syringe pump. For testing, the microdialysis probes were mounted in a beaker to which 22.5 mL of simulated intestinal media were added (see below). The media were stirred at 300 rpm and kept at 37 °C by using a magnetic stirrer with heating. The microdialysis probes were perfused with 2% PS80 in lipolysis buffer at a rate of 5 μL/min. These settings were previously identified as the optimal conditions for microdialysis sampling of indomethacin yielding recoveries of approximately 80% and fairly rapid response times of 10 min.^23^ Before and after each experiment, the probe specific recoveries were determined using an indomethacin solution with a low and a high concentration (i.e., 2 and 100 μg/mL), to account for day-to-day recovery differences and to ensure the experiment had not impacted the probes in any way. The probe specific recovery (eq 1) is the ratio of the indomethacin concentration in the dialysate (*C*_*D*_) compared to the indomethacin in the bulk solution (*C*_*S*_):

$$recovery=\frac{C_{D}}{C_{S}}\cdot 100\%$$ 1

It needs to be mentioned that upon dissolution in lipolysis buffer containing calcium, indomethacin forms very small nanoparticles (particle size <2 nm) that enhanced indomethacin’s permeation across a biomimetic barrier,^23^ which is in line with enhanced bioavailability of indomethacin calcium.^31^ These very small nanoparticles can pass the 100 kDa MWCO microdialysis probes (but not those with a 20 kDa cutoff, which can be regarded as the standard pore size), and hence, it is expected that in all microdialysis samples, indomethacin is present in the form of very small nanoparticles. In contrast, flow field flow fractionation studies of micelles in aspirated human intestinal as well as artificial intestinal fluids indicate micelle sizes of approximately 30 nm and above^32^ and are thus not expected to penetrate. The fraction of indomethacin that passes through the 100 kDa MWCO microdialysis probes, in the form of very small nanoparticles and/or molecularly dissolved indomethacin (i.e., indomethacin surrounded by a hydration shell), will forth on be referred to as the ‘free fraction’ of indomethacin. The free fraction was calculated by applying the recovery (%*R*) as a correction factor according to eq 2:

$$free\;fraction=\frac{C_{D}}{\left(\frac{\%R}{100}\right)}$$ 2

#### 2.6.1 Microdialysis Sampling in Simulated Intestinal Media with Mixed Bile Salt-Phospholipid Micelles

To demonstrate that microdialysis sampling can discern the free fraction of indomethacin from indomethacin associated with mixed bile salt-phospholipid micelles, microdialysis sampling of indomethacin suspended in three simulated intestinal media was conducted as described by Holzem et al.^27^ The biomimetic media were prepared as described in Section 2.2 and contained 1.5 mM sodium taurocholate and 0.375 mM lecithin, 3 mM sodium taurocholate and 0.75 mM lecithin, 15 mM sodium taurocholate, and 3.75 mM lecithin. These concentrations correspond to 0.5, 1 and 5-times the amount of FaSSIF/FeSSIF/FaSSGF powder recommended by the manufacturer, respectively. The latter corresponds to the concentrations found in fed state intestinal fluids. The indomethacin suspensions (1.556 mg/mL) were prepared by suspending 35 mg indomethacin in 22.5 mL simulated intestinal medium or lipolysis buffer. The suspensions were incubated at 37 °C overnight in a shaking water bath to ensure equilibrium.

The microdialysis experiments were started by inserting the three microdialysis probes into three separate beakers each containing a preheated (37 °C) indomethacin suspension in lipolysis buffer (i.e., a sample without mixed micelles). Microdialysis sampling was performed by constant perfusion of the probes, and dialysate samples were collected directly into HPLC vials with inserts at *t* = 0–5, 5–10, 20–25, and 55–60 min. The collected dialysate was diluted with HPLC mobile phase prior to HPLC-UV analysis (dilution factor 4). As a comparison, samples were taken manually and processed in a conventional way by centrifugation at *t* = 0, 5, 10, 25, and 60 min (i.e., referred to as ‘conventional samples’). For this, 125 μL were transferred to microcentrifuge tubes and immediately centrifuged at 14,000 rpm (24,104 × g) for 15 min at 37 °C using the Centrifuge 5804 R with the F-45-30-11 rotor (Eppendorf AG, Hamburg, Germany). Fifty μL of the supernatant was diluted with HPLC mobile phase prior to HPLC-UV analysis (dilution factor 3). After 60 min, the microdialysis probes were placed in the simulated intestinal medium with 1.5 mM sodium taurocholate and 0.375 mM lecithin, and the procedure was repeated collecting microdialysis and conventional samples. The procedure was repeated twice more, placing the microdialysis probes in the simulated intestinal media with 3 mM sodium taurocholate and 0.75 mM lecithin and 15 mM sodium taurocholate and 3.75 mM lecithin, respectively.

#### 2.6.2 Microdialysis Sampling in Simulated Intestinal Medium with Pancreatin Extract

The experiments exploring the compatibility of the microdialysis probes with pancreatin extract were based on the standard in vitro lipolysis method described by Williams et al. (^10^). An indomethacin suspension in lipolysis medium (1.556 mg/mL) was prepared as described above (Section 2.6.1). As described by Hedge et al. (^30^), the lipolysis buffer used to prepare the lipolysis media contained 200 mM tris-maleate in order to maintain pH within 0.3 units of the initial value, despite the release of free fatty acids, since no (auto)­titration was used in the experiments. Pancreatin extract was prepared by dispersing 200 mg/mL porcine pancreatin in 3 mL of cold (2–8 °C) lipolysis buffer, followed by centrifugation at 5 °C and 3,000 rpm (1107 × g) for 15 min (Centrifuge 5804 R, rotor type: F-45-30-11 Eppendorf AG, Hamburg, Germany). The supernatant is the pancreatin extract, which was prepared freshly before each experiment to minimize the loss of enzyme activity prior to use. The experiment was started by inserting the three microdialysis probes into three separate beakers each containing 20 mL of preheated (37 °C) indomethacin suspension in lipolysis medium and adding 2.5 mL of pancreatin extract. The sampling procedures and sample preparation were identical to the one used in the experiment described in Section 2.6.1, At the end of the experiment, the pH of the indomethacin suspension was measured.

### 2.7 Microdialysis Sampling during In Vitro Lipolysis

In vitro lipolysis experiments were based on the standard procedure described by Williams et al.^10^ The experiments were performed in a simple temperature controlled (37 °C) beaker setup with magnetic stirring (300 rpm), similar to the one used for microdialysis sampling (Section 2.6). Twenty mL of the lipolysis medium (preheated to 37 °C) and 667 mg medium-chain type IIIB LBF loaded with indomethacin (preheated to 37 °C) were added to the beaker and allowed to mix at 300 rpm for 10 min (i.e., dispersion phase). Lipolysis was initiated by adding 2.5 mL of pancreatin extract, which was prepared as described above (Section 2.6). During lipolysis, samples were taken conventionally as well as through microdialysis. For conventional sampling, samples of 125 μL were taken at *t* = −5 min (i.e., equals to 5 min of the dispersion phase), 0 min (i.e., immediately after addition of the pancreatin extract), and 5, 10, 15, 20, 25, 30, 35, 40, 45, 60, 90, 120, 150, and 180 min. These samples were immediately centrifuged at 14,000 rpm (24,104 × g) and 37 °C for 15 min (Centrifuge 5804 R, rotor type: F-45-30-11 Eppendorf AG, Hamburg, Germany). In this case, the samples were separated into a solid pellet phase, a particle-free supernatant, and an oil phase. As described above, 50 μL of the aqueous phase was diluted with HPLC mobile phase. Microdialysis sampling was performed by constant perfusion of the probes, starting at the beginning of the dispersion phase, and dialysate samples were collected directly into HPLC vials with inserts at *t* = 0–5, 5–10, 10–15, 15–20, 20–25, 25–30, 30–35, 35–40, 40–45, 55–60, 85–90, 115–120, 145–150 and 175–180 min. As described above, the dialysate was diluted with HPLC mobile phase prior to HPLC analysis. At the end of the experiment, the pH of the digestion medium was measured to ensure that it stayed within 0.3 units of the initial value, despite the release of free fatty acids.

To better understand how digestion influenced the performance of the LBF, two control experiments were conducted. First, an experiment was conducted where 42 mg of crude indomethacin was added to the reaction vessel instead of the LBF. Otherwise, the experiment was similar to the one with the LBF. Second, an experiment without the addition of pancreatin extract to the reaction vessel was conducted. In this experiment, the LBF was dispersed in lipolysis medium, but due to the absence of pancreatin extract, the LBF was not digested. Hence, this experiment can be regarded as a simple dissolution or dispersion experiment under nonlipolytic conditions. All experiments were performed in triplicate.

### 2.8 Statistics and Data Analysis

All data handling, visualization and statistical analysis was carried out in Microsoft Excel. An unpaired parametric student’s *t* test was used to evaluate whether there were statistically significant differences between conventional samples and microdialysis samples when sampling from simulated intestinal media without pancreatin. A p-value of <0.05 was considered as significant. All values are expressed as the mean ± SD of the independent replicates, with a minimum of 3 replicates for each performed experiment.

## 3 Results and Discussion

### 3.1 Solubility

The equilibrium solubility (*S_eq_*) of indomethacin in lipolysis medium (i.e., lipolysis buffer with 3 mM sodium taurocholate and 0.75 mM lecithin) was 1,051 ± 39.58 μg/mL. Previously, the *S_eq_* of indomethacin in lipolysis buffer with the same pH of 6.5 was determined to be 490.6 ± 17.85 μg/mL.^23^ Hence, the presence of mixed micelles increased indomethacin’s solubility 2.1-fold compared to the corresponding medium without bile salts and lecithin (i.e., lipolysis buffer). This shows that indomethacin was solubilized by mixed bile salt-phospholipid micelles to a significant extent.

### 3.2 Compatibility of Microdialysis Sampling with Simulated Intestinal Conditions

#### 3.2.1 Mixed Bile Salt-Phospholipid Micelles

To study if microdialysis sampling can discern the free fraction of indomethacin from indomethacin associated with mixed bile salt-phospholipid micelles, microdialysis sampling was performed on indomethacin suspensions in media containing varying amounts of sodium taurocholate and lecithin. For all microdialysis results presented, calibration of the probes was conducted as described in Section 2.6. An example of the calibration (i.e., determination of the probe specific recovery) is given in the Supporting Information (Figure S1). As a comparison to microdialysis, samples were taken in a conventional way. The results are presented in Figure 1 showing concentration versus time profiles for the different indomethacin suspensions. For all experiments, the pH measured at the end was equal to the one measured before the experiment (i.e., pH = 6.50).

**Figure 1:**
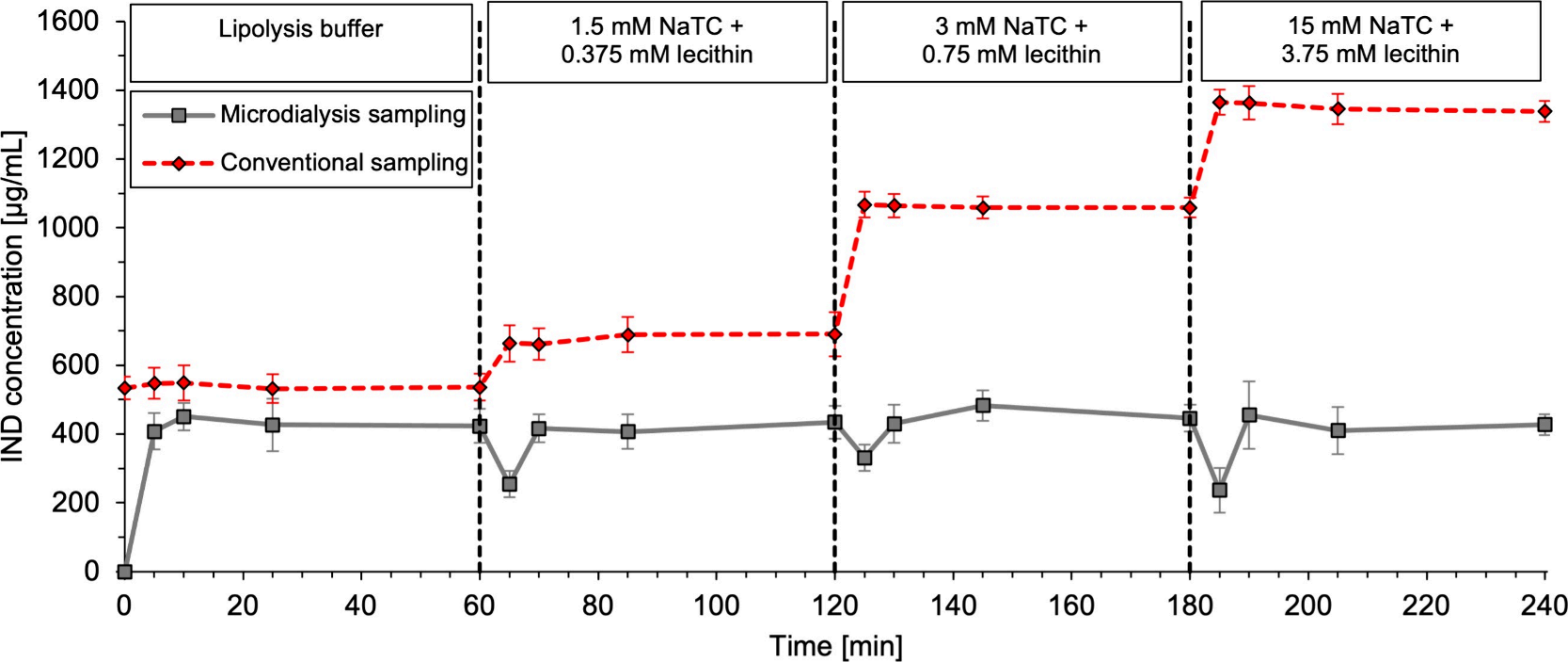
Indomethacin (IND) concentration vs time profiles for indomethacin (1.6 mg/mL) suspended in lipolysis buffer or simulated intestinal media with differing amounts of sodium taurocholate and lecithin. The first, second, and third vertical dashed lines represent medium changes from lipolysis buffer to simulated intestinal medium with 1.5 mM sodium taurocholate (NaTC) and 0.375 mM lecithin, 3.0 mM NaTC and 0.75 mM lecithin, and 15 mM NaTC and 3.75 mM lecithin, respectively. Concentrations were determined following conventional sampling (red diamonds) and microdialysis sampling (gray squares). Data is presented as the mean ± SD, *n* = 3.

When indomethacin was suspended in lipolysis buffer (i.e., a control without any mixed micelles), the concentration of the samples taken conventionally demonstrated a constant concentration equivalent to *S_eq_* in the lipolysis buffer. After the expected stabilization latency of 10 min, microdialysis sampling, as well, displayed stable concentrations though around 20% lower than the concentration of the conventional samples. However, when performing statistical analysis between the microdialysis and conventional sample data points (after the 10 min latency time), there is no statistical difference between the two sampling methods (*p* > 0.05). Even though there was no statistical difference between the two sampling methods, we cannot exclude there may be some minor systematic differences between the methods considering their different separation principles. The most important difference is though the effect of the mixed micelles as described below.

After the microdialysis probes were transferred to the indomethacin suspension in simulated intestinal medium with 1.5 mM sodium taurocholate and 0.375 mM lecithin, the indomethacin concentration in the conventional samples increased noticeably to approximately 670 μg/mL due to micellar solubilization. In contrast, the microdialysis samples, after the expected stabilization latency of 10 min, displayed stable concentrations identical to those observed in the absence of mixed micelles. When increasing the sodium taurocholate and lecithin concentration in the medium to 3 mM and 0.75 mM, and thereby the number of mixed micelles, similar observations were made; the concentration of the conventional samples increased to approximately 1100 μg/mL corresponding to the *S_eq_* of indomethacin in lipolysis medium, and the concentration of the microdialysis samples remained the same (after the 10 min stabilization latency). Further increasing the sodium taurocholate and lecithin concentration in the medium to 15 and 3 mM, increased the indomethacin concentration in the conventional samples to approximately 1350 μg/mL whereas the concentration in the microdialysis samples still remained at 480 μg/mL, similar to all other media.

These results clearly demonstrate that microdialysis sampling could distinguish between the free fraction of indomethacin and indomethacin associated with mixed micelles regardless of supplementation with different amounts of bile salt and phospholipid. These findings agree with previous in vitro microdialysis findings, which demonstrated that microdialysis sampling can distinguish between freely dissolved posaconazole and posaconazole associated with mixed micelles.^27^

#### 3.2.2 Pancreatin Extract

To further study the compatibility of microdialysis sampling with in vitro lipolysis conditions, microdialysis sampling was performed in the presence of pancreatic enzymes. Figure 2 shows indomethacin concentration vs time profiles when sampling an indomethacin suspension in lipolysis medium supplemented with pancreatin extract. The pH measured at the end of the experiment (i.e., 6.497 ± 0.0047) was equal to the one measured before the experiment (i.e., pH = 6.50). For comparison, concentration vs time profiles for indomethacin suspensions in lipolysis buffer and lipolysis media without pancreatin extract are also shown.

**Figure 2:**
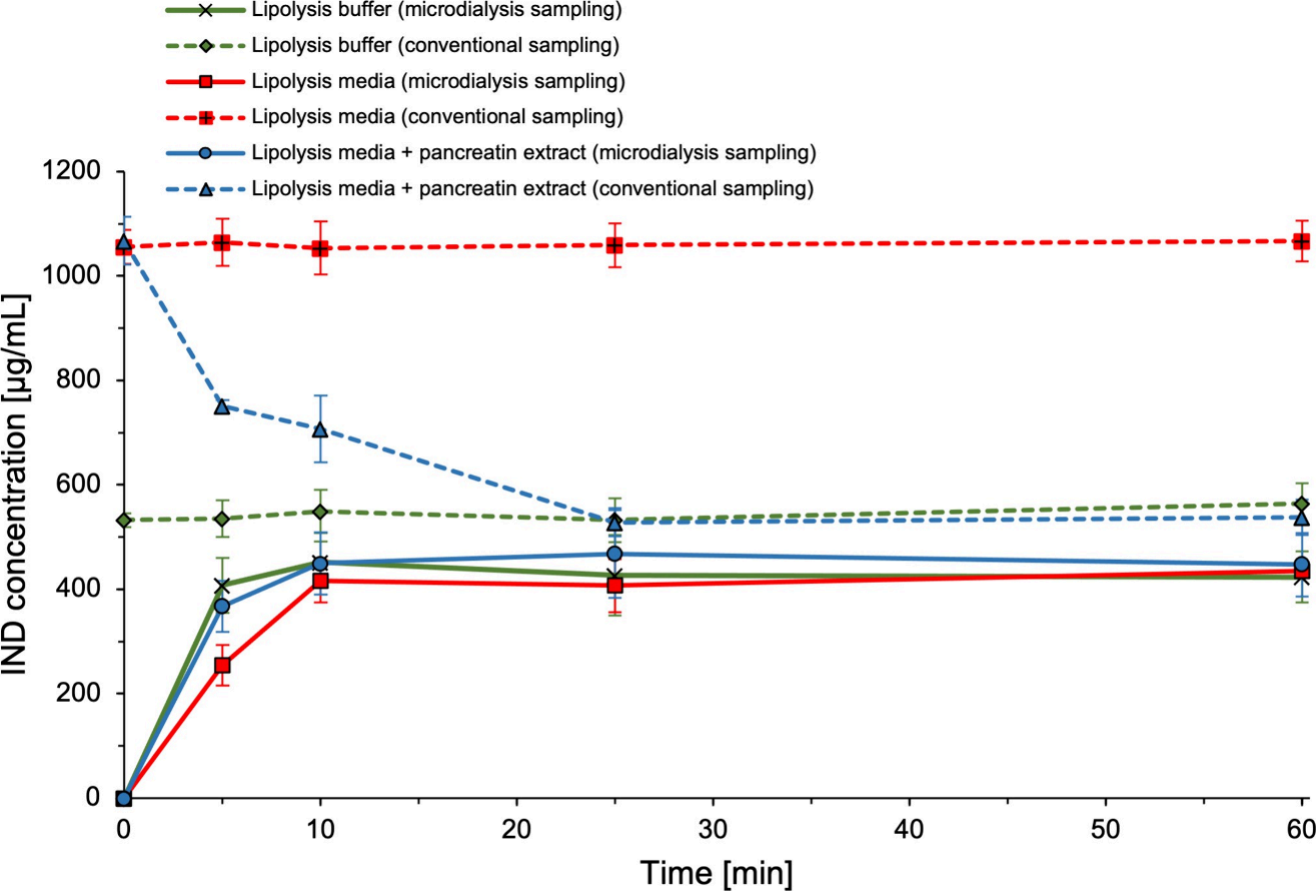
Indomethacin (IND) concentration vs time profiles for indomethacin (1.6 mg/mL) suspended in lipolysis buffer (green symbols), lipolysis media (red symbols), and lipolysis media with pancreatin extract (blue symbols). Concentrations determined following conventional sampling (dashed lines) and microdialysis sampling (solid lines). Data is presented as mean ± SD, *n* = 3.

In the beginning of the experiment (i.e., at *t* = 0), conventional samples of indomethacin suspended in lipolysis medium supplemented with pancreatin extract demonstrated concentrations equal to those measured in lipolysis medium, thereby matching the *S_eq_* of indomethacin in lipolysis medium. Upon beginning of the lipolysis procedure, due to the presence of pancreatin extract, the indomethacin concentration in the conventional samples quickly decreased within the first five min, whereafter the concentration reached *S_eq_* of indomethacin in lipolysis buffer within 25 min. This drop in concentration can be explained by the enzymatic breakdown of the solubilizing mixed micelles, which are present in the lipolysis medium. In more detail, pancreatin extract contains phospholipases (e.g., phospholipase A2), which cleave phospholipids,^33^ whereby the micellar structures created by phospholipids and bile salts are extinguished resulting in precipitation of indomethacin.

In contrast, microdialysis samples, after the expected stabilization latency of 10 min, displayed stable concentrations equal to those in lipolysis buffer and lipolysis medium throughout the remaining 50 min of the experiment. These results demonstrate that microdialysis sampling of indomethacin is possible in the presence of pancreatin extract, which is crucial for its use for in vitro lipolysis.

### 3.3 Microdialysis Sampling during In Vitro Lipolysis and Dissolution of a Lipid-Based Formulation Loaded with Indomethacin

To test microdialysis sampling of indomethacin during in vitro lipolysis, a medium-chain type IIIB LBF was prepared according to Alskär et al.^29^ This LBF was tested by in vitro lipolysis as well as dissolution/dispersion without addition of pancreatic enzymes in order to see the impact of digestion on the performance of the formulation. In both cases, sampling was performed both conventionally and through microdialysis. Additionally, crude indomethacin, in the same dose as that loaded in the LBF, was used as a control. The in vitro lipolysis and dissolution results for the LBF and for crude indomethacin are given in Figure 3. The pH measured at the end of the LBF dissolution and lipolysis experiments (i.e., pH 6.49 ± 0.0082 and 6.49 ± 0.0047, respectively) was equal to the one measured before the experiment (i.e., pH 6.50), indicating sufficient buffer capacity also in the presence of the formulation.

**Figure 3:**
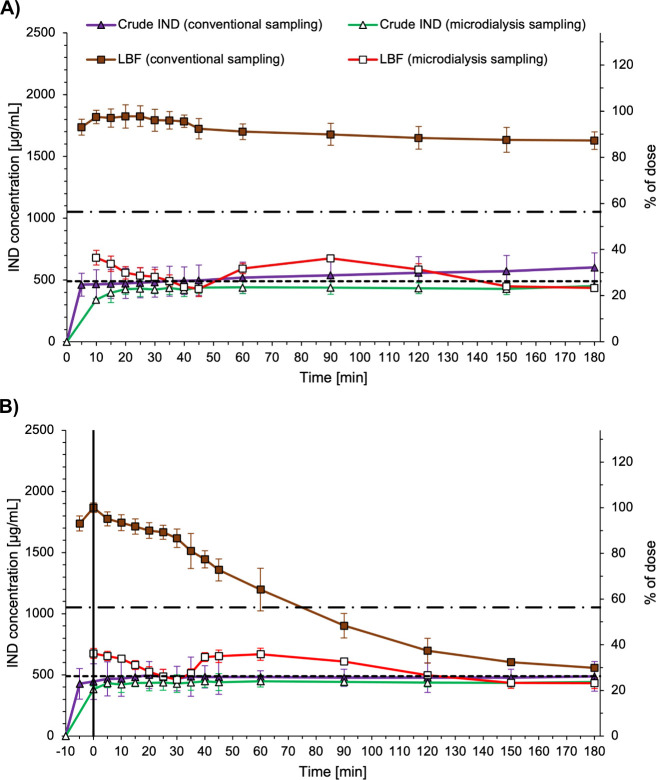
Indomethacin concentrations during dissolution/dispersion (A) and in vitro lipolysis (B) of the medium-chain type IIIB LBF with indomethacin (squares) and crude indomethacin as a control (triangles). Samples were taken conventionally (open symbols) or via microdialysis (filled symbols). Horizontal dashed line represents *S_eq_* of indomethacin in lipolysis buffer from Tønning et al. (^23^), and horizontal line of alternating dashes and dots represents *S_eq_* of indomethacin in lipolysis medium. In part B, the solid vertical line indicates the end of the 10 min dispersion phase and subsequent addition of pancreatin extract starting the digestion. All data are presented as the mean ± SD, *n* = 3.

Figure 3a shows concentration vs time profiles for the dissolution (dispersion) of the LBF loaded with indomethacin in lipolysis media, as well as the control (crude indomethacin). During the three-hour dissolution experiment, the control (crude indomethacin) indicated the expected dissolution behavior of indomethacin in lipolysis medium. In detail, the conventional samples confirmed that crude indomethacin dissolved slowly and incompletely over time and subsequently reached concentrations slightly higher than the *S_eq_* of indomethacin in pure lipolysis buffer with a final concentration of 602.0 μg/mL. Slightly higher concentrations than the *S_eq_* of indomethacin in pure lipolysis buffer are due to micellar solubilization. Presumably, with a longer dissolution time, the concentration would reach the *S_eq_* of indomethacin in lipolysis medium. Simultaneously, the concentration of the microdialysis samples did not go beyond the *S_eq_* of indomethacin in pure lipolysis buffer, which again demonstrated that microdialysis is able to discriminate between the free fractions of indomethacin and indomethacin associated with mixed micelles.

As expected, during the three-hour dissolution/dispersion of the LBF, the indomethacin concentrations of the conventional samples were much higher than those during dissolution of crude indomethacin. The LBF demonstrated an impressive solubilization capacity for indomethacin, ensuring almost complete solubilization of the dose during the three h dissolution experiment. This agrees with the study by Alskär and co-workers, who reported a solubility of indomethacin of 1.5–1.7 mg/mL in the different dispersed LBFs.^29^ In more detail, indomethacin was fully solubilized until approximately 40 min before the concentration very slowly declined, likely due to precipitation. Still, close to 90% of the dose remained solubilized at the end of the experiment in conventional samples. From this conventional sampling approach, the performance of the LBF under nonlipolytic conditions can be regarded as excellent.

Interestingly, the concentration determined via microdialysis sampling indicated substantially different behavior. Here, the indomethacin concentration started at approximately 700 μg/mL, which was clearly above the *S_eq_* of indomethacin in lipolysis buffer, but much lower than the concentration of the conventional samples indicating almost complete solubilization. This means that a significant amount of indomethacin was solubilized by colloidal structures that cannot pass the microdialysis membrane. This means also that, at the start of the experiment, the dissolved indomethacin (700 μg/mL), passing the microdialysis membrane, must be in a supersaturated state with regard to the equilibrium solubility of indomethacin in pure lipolysis buffer (490 μg/mL). From these results, it is clear that the formulation acted as a supersaturation-inducing “spring” when dispersed in the lipolysis medium.

After the initial supersaturation, the concentration steadily declined before it reached the *S_eq_* of indomethacin in lipolysis buffer at the 45 min sampling time point. Surprisingly, a second phase with supersaturated indomethacin concentrations was observed between 60 and 150 min. With the current data, this phenomenon is difficult to explain. However, hypothetically, the second supersaturation phase may be caused by the rearrangement of colloidal assemblies in the dissolution medium, which contains bile salts and phospholipids. Bile salts adsorb to lipid droplets^34^ and thereby play a role in their emulsification in the intestine.^35^ Hypothetically, emulsification of the lipid droplets formed by dispersion of the LBF could have happened here, causing a possible rearrangement and thereby a possible decline in the solubilization capacity for indomethacin. A decline in solubilization capacity could lead to the release of indomethacin from colloidal assemblies, causing a supersaturated state, which can be detected in the microdialysis samples but not in the conventional samples. However, additional experiments are required to investigate this hypothesis. Here, the colloidal ultrastructure should be followed during dispersion/dissolution, where a combination of different techniques may be required.^36^

Figure 3b shows concentration vs time profiles during in vitro lipolysis of the LBF and crude indomethacin as the control. During the three-hour in vitro lipolysis experiment, crude indomethacin followed the expected behavior resembling the behavior during the dissolution experiment with slight differences. In more detail, the conventional samples again indicated that the crude indomethacin dissolved slowly and incompletely over time. However, in the lipolysis experiment, the concentration did not exceed the *S_eq_* of indomethacin in pure lipolysis buffer as observed during dissolution. This is likely due to the previously explained cleavage of phospholipids by phospholipase A2 present in pancreatin extract, which disturbs the solubilizing mixed micelles in the lipolysis medium.^33^ In line with this, the free indomethacin concentration measured via microdialysis sampling did not exceed the *S_eq_* of indomethacin in pure lipolysis buffer either.

Similar to the dissolution experiment, the LBF again exhibited an impressive solubilization capacity for indomethacin during the 10 min dispersion phase, showing complete solubilization. After the addition of the pancreatin extract, initiating the digestion of the formulation, indomethacin concentrations in the conventional samples steadily declined. These concentrations decreased below the *S_eq_* of indomethacin in lipolysis medium at the 90 min sampling point and were close to the *S_eq_* of indomethacin in lipolysis buffer at the final sampling point. This can be attributed to the enzymatic breakdown of the LBF, which results in precipitation of indomethacin due to a loss of the solubilizing effects achieved by the formulation. Based on the conventional samples, lipolysis seems to affect the performance of the LBF negatively; i.e., the solubilization capacity is lost with digestion, which conventionally would be regarded as detrimental.

Alskär et al. reported only a small decrease of indomethacin solubility in medium with the digested LBFs compared to medium with the dispersed LBFs (i.e., from 1.5 to 1.7 to 1.1 mg/mL). In Alskär’s study, the LBFs were digested only 60 min before solubility of indomethacin was determined.^29^ Referring to Figure 3b, the indomethacin concentration of the conventional samples at 60 min is approximately 1.2 mg/mL, which agrees with Alskär et al. A significant drop in solubilization capacity was observed after 60 min of digestion, highlighting also the importance of the assay time when evaluating LBF performance. For reference, the intestinal residence time is on average 3–4 h.^37^

Surprisingly, the indomethacin concentrations in the microdialysis samples during in vitro lipolysis of the LBF were nearly identical to the concentrations measured during dissolution without the addition of enzyme. Again, indomethacin was supersaturated in the beginning of the experiment with the formulation acting as a “spring”, and again, the concentration declined to *S_eq_* of indomethacin in lipolysis buffer. Also in this experiment, a surprising second supersaturation phase was observed. Though, in this case, the second supersaturation phase started slightly earlier with slightly higher concentrations. Also in this case, the data currently available cannot explain this phenomenon. Similarly to what is described above for the dissolution/dispersion of the LBF, the second supersaturation phase may be caused by changes in the colloidal ultrastructure, where in this case also lipolysis may be an influencing factor by breaking down formulation constituents. The products can be amphiphilic (e.g., fatty acids and monoglycerides). With scattering techniques, such changes of the colloidal structures during digestion of LBFs have been shown.^38,39^ With small-angle X-ray scattering (SAXS), the formation of a vesicular phase was observed during digestion of a medium-chain LBF dependent on lipid mass and bile salt concentration.^38^ With small-angle neutron scattering (SANS), micellar elongation was observed during digestion of oleic acid glycerides due to incorporation of digestion products, monoglycerides and fatty acids, into the micelles.^39^

In summary, depending on which sampling technique is used, two conflicting conclusions about the performance of the LBF under lipolytic conditions can be drawn. Either the performance of the LBF is affected negatively by digestion (as indicated by conventional sampling) or, in contrast, the performance is not affected by digestion (as indicated by microdialysis sampling).

As an essential physiological process, the influence of digestion on the performance of LBFs should be considered as highlighted in a recent review.^40^ Taking this important factor into account, previous studies have compared the performance of LBFs under lipolytic or nonlipolytic conditions comparing in vitro and in vivo results using cinnarizine,^7^ fenofibrate,^41^ and halofantrine^42^ as model drugs. Nonlipolytic conditions in vivo are typically achieved by simultaneously administering a lipase inhibitor (e.g., orlistat). For fenofibrate and halofantrine in self-emulsifying drug delivery systems, precipitation during dynamic in vitro lipolysis was significantly lower under nonlipolytic conditions (i.e., with orlistat). In these in vitro studies, samples were taken in a conventional manner, involving centrifugation for separation. These observations are similar to our observations with indomethacin (i.e., the indomethacin concentration in the conventional samples was much higher under nonlipolytic conditions as compared to lipolytic conditions). In contrast, halofantrine and fenofibrate absorption in vivo was generally not affected by lipase inhibition.^41,42^ In an in vivo study in rats it was demonstrated that digestion had little to no impact on the bioavailability of cinnarizine in a medium-chain type IIIB LBF.^8^ These previous studies agree with what we observed in this study. Namely, the performance of an LBF can be independent of digestion (as indicated by microdialysis samples), even though digestion causes a loss of solubilization capacity (as indicated by conventional samples). Although different model compounds were used, the in vivo results may indicate that microdialysis sampling could help to assess the performance of LBFs in a meaningful way as compared to conventional sampling, which is usually used during in vitro lipolysis for a performance ranking of LBFs.

Furthermore, an important advantage of microdialysis sampling over conventional sampling is that, due to the perfusion principle, samples are not taken directly from the reaction vessel, keeping the disturbance of the system to a minimum. In contrast, during conventional sampling with benchtop centrifugation for separation, a significant sample volume is removed from the reaction vessel (e.g., typically 0.5–1 mL per sample). An even bigger sample volume can be required for ultracentrifugation (e.g., 4 mL), which has traditionally been used for separation of in vitro lipolysis samples.^10^ For many in vitro lipolysis samples, except those with an oil-rich phase, benchtop centrifugation yields comparable results to ultracentrifugation,^10^ which is why benchtop centrifugation is conventionally used today considering its higher availability and lower cost.

A potential advantage of ultracentrifugation, like microdialysis sampling, may be the possibility to separate colloid-associated drug molecules from molecularly dissolved drug molecules. To the best of our knowledge, this has not been attempted with pharmaceutical in vitro lipolysis samples but would likely require ultracentrifugation of the isolated aqueous micellar phase. Though possible, the separation of small colloidal assemblies such as micelles is not easy. For example, polysorbate 80 micelles were separated from a solution of poorly soluble estradiol by ultracentrifuging at 451,268 g (100,000 rpm) for 4 h.^43^ The time aspect with centrifugation durations of several hours is a disadvantage itself. Additionally, long centrifugation times may lead to artifacts since in vitro lipolysis samples are not in equilibrium (e.g., transient supersaturation may collapse during separation). Here, microdialysis sampling has a clear advantage, being almost instantaneous (i.e., after collection of a sample fraction over typically 5–10 min, the sample is analyzed). Another disadvantage of ultracentrifugation may be the high forces that act on the “soft” lipolysis assemblies: Those could be disrupted, and the entrapped drug could be released. In contrast, microdialysis is gentle. A common disadvantage of ultracentrifugation and microdialysis sampling is the possibility of drug absorption into either centrifuge tubes or microdialysis tubing. Here, presaturation can be a remedy for both techniques. Despite many positive features, microdialysis sampling has one important challenge: microdialysis is useful only after careful calibration of the probes. For this, a solution of the analyte is required, which is difficult to prepare in the case of very poorly soluble drugs.

When using conventional sampling with benchtop centrifugation or short ultracentrifugation for separation of the aqueous phase during in vitro lipolysis for performance ranking of LBFs, an in vitro-in vivo mismatch has been observed on several occasions, and the need for an alternative method for performance ranking has been voiced.^9,12^ An example of an alternative method is the combination of in vitro lipolysis and permeation, which has proven to be useful for performance assessment of LBFs.^15,17,21^ However, in vitro lipolysis combined with permeation does not provide real-time information about the freely dissolved fraction during in vitro lipolysis. Rather, it can be viewed as a “black box experiment”, where the (unknown) free fraction determines the permeation rate, which is quantified and used for the LBF performance ranking.

Microdialysis sampling, on the other hand, due to its fast response time, gives almost real-time information about the free fraction, thereby yielding possible mechanistic explanations for permeation or even oral absorption under lipolytic conditions. It is worth mentioning, however, that the scenario that governs drug absorption is expected to be far more complex and dynamic. We are currently unable to delineate the individual kinetics of the manyfold underlaying elementary processes potentially governing drug absorption including release, dissolution, supersaturation, micelle association and release, precipitation, and permeation. Few researchers have come up with experimental approaches to determine the association- release- and transfer-kinetics of poorly water-soluble drugs to/from colloidal assemblies.^44–46^

## 4 Conclusions

In this study, the feasibility of microdialysis sampling during in vitro lipolysis was tested. Indomethacin was used as a model drug, which was sampled using a previously developed method consisting of 100 kDa MWCO microdialysis probes perfused with 2% PS80 in lipolysis buffer at a rate of 5 μL/min.^23^ Compatibility of microdialysis sampling with in vitro lipolytic conditions was tested by sampling indomethacin from simulated intestinal media with different bile salt and phospholipid concentrations and by sampling indomethacin from simulated intestinal media with pancreatic enzymes. In all simulated intestinal media, microdialysis sampling clearly differentiates the free fraction of indomethacin from the fraction associated with mixed micelles. Most importantly, microdialysis sampling was possible in the presence of pancreatic extract, a crude material containing pancreatic lipases, which are essential for the conventional in vitro lipolysis protocol.

As a proof-of-concept, indomethacin was loaded in a medium-chain type IIIB LBF, and the release was tested under lipolytic and nonlipolytic conditions using both conventional sampling and microdialysis sampling for comparison. Interestingly, different conclusions about the performance of the LBF under lipolytic conditions could be drawn depending on which samples were used as the reference. The conventional samples indicated that lipolysis was detrimental to the performance of the LBF by reducing its solubilization capacity. Microdialysis samples, however, indicated that the performance was not affected by lipolysis, showing that the LBF acted as a supersaturation-inducing spring in both cases. Using other model drugs, previous in vivo studies have shown LBF performance to be independent of lipolysis, even though in vitro lipolysis experiments (with conventional sampling) indicated otherwise. In line with in vivo observations, this study indicates that microdialysis sampling could be a useful and complementary sampling technique for assessing the performance of LBFs. Future studies should include other drugs and other types of LBFs to show a broader applicability of microdialysis sampling during in vitro lipolysis, by which potentially LBF development could be significantly improved.

## Supplementary Material



## Notes

Figure S1: Representative calibration of three microdialysis probes as microdialysis recovery vs time profiles (PDF)

## Abbreviations

IND: indomethacin
LBF: lipid-based formulation
LCFS: Lipid Formulation Classification System
MWCO: molecular-weight-cutoff
PS80: polysorbate 80
SAXS: small-angle X-ray scattering
SANS: small-angle neutron scattering
*S_eq_*: equilibrium solubility
