# Supplementary material for: Looking behind the Solubilization Curtain: Microdialysis Provides Near-Real-Time Noncolloidal Drug Concentrations during In Vitro Lipolysis
Source: Mol Pharm. 2025 Oct 3;22(11):6670–80. doi: 10.1021/acs.molpharmaceut.5c00640 (PMC12587442; doi:10.1021/acs.molpharmaceut.5c00640)
Supplement: Supplementary file 1 [file mp5c00640_si_001.pdf]

Supplementary material

Looking behind the solubilization curtain: Microdialysis provides near real-time non-colloidal drug concentrations during in vitro lipolysis

Mikkel Højmark Tønning [a], Annette Bauer-Brandl [a], Martin Brandl [a], Felix Paulus [a], Ann-Christin Jacobsen [b*]

[a] Department of Physics, Chemistry & Pharmacy, University of Southern Denmark, 5230 Odense, Denmark
[b] Department of Pharmaceutics and Biopharmaceutics, Kiel University, 24118 Kiel, Germany

* Email: ajacobsen@pharmazie.uni-kiel.de

S1: recovery

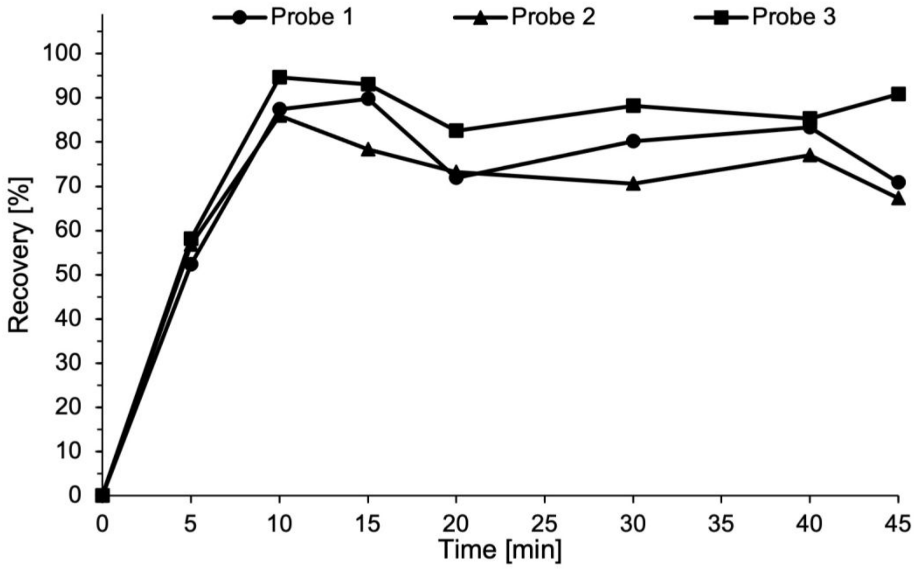

Figure S1 Representative calibration of three microdialysis probes as microdialysis recovery vs. time profile. At t = 0, 100 kDa cut-off microdialysis probes were placed in an indomethacin solution in lipolysis buffer (100 µg/mL). Probes were perfused for 45 min. The perfusion flow was 5 µL/min, and the perfusion medium was lipolysis buffer with 2% (v/v) polysorbate 80.
